# The role of PTEN - HCV core interaction in hepatitis C virus replication

**DOI:** 10.1038/s41598-017-03052-w

**Published:** 2017-06-16

**Authors:** Qi Wu, Zhubing Li, Paul Mellor, Yan Zhou, Deborah H. Anderson, Qiang Liu

**Affiliations:** 1 Vaccine and Infectious Disease Organization-International Vaccine Center and Department of Veterinary Microbiology, University of Saskatchewan, Saskatoon, Saskatchewan Canada; 2 Vaccine and Infectious Disease Organization-International Vaccine Center and School of Public Health, University of Saskatchewan, Saskatoon, Saskatchewan Canada; 3 Cancer Research Group, University of Saskatchewan and Saskatchewan Cancer Agency, Saskatoon, Saskatchewan Canada; 4 Vaccine and Infectious Disease Organization-International Vaccine Center, Department of Veterinary Microbiology and School of Public Health, University of Saskatchewan, Saskatoon, Saskatchewan Canada

## Abstract

Hepatitis C virus (HCV) infection leads to severe liver diseases including hepatocellular carcinoma (HCC). Phosphatase and tensin homolog deleted on chromosome 10 (PTEN), a tumour suppressor, is frequently mutated or deleted in HCC tumors. PTEN has previously been demonstrated to inhibit HCV secretion. In this study, we determined the effects of PTEN on the other steps in HCV life cycle, including entry, translation, and replication. We showed that PTEN inhibits HCV entry through its lipid phosphatase activity. PTEN has no effect on HCV RNA translation. PTEN decreases HCV replication and the protein phosphatase activity of PTEN is essential for this function. PTEN interacts with the HCV core protein and requires R50 in domain I of HCV core and PTEN residues 1–185 for this interaction. This interaction is required for PTEN-mediated inhibition of HCV replication. This gives rise to a reduction in PTEN levels and intracellular lipid abundance, which may in turn regulate HCV replication. HCV core domain I protein increases the lipid phosphatase activity of PTEN in an *in vitro* assay, suggesting that HCV infection can also regulate PTEN. Taken together, our results demonstrated an important regulatory role of PTEN in the HCV life cycle.

## Introduction

More than 185 million people are estimated to be infected by hepatitis C virus (HCV) worldwide^1^, leading to severe liver diseases such as hepatocellular carcinoma (HCC). HCV is an enveloped positive-sense single-stranded RNA virus in the *Flaviviridae* family^2^. HCV life cycle begins with cell entry through cellular receptors. The HCV genomic RNA is then translated into a polyprotein through an internal ribosomal entry site (IRES)^3^. The polyprotein is processed by cellular and viral proteases to generate structural (core, E1, and E2) and non-structural (p7, NS2–5B) proteins. The endoplasmic reticulum membrane is modified by viral and cellular factors to generate a membranous web that is the major site of viral RNA replication. Viral RNAs are packaged into nucleocapsids followed by particle maturation and secretion after envelope acquisition. Although it is the nucleocapsid protein, HCV core has many regulatory functions^4^, and is associated with the development of HCC in a transgenic mouse model^5^.

Phosphatase and tensin homolog deleted on chromosome 10 (PTEN) is a phosphatase with both lipid and protein phosphatase activities^6^. PTEN contains an N-terminal PtdIns(4,5)P_2_-binding domain (aa. 1–6), a phosphatase domain (aa. 7–185), a C2 domain (aa. 186–351), and a C-terminal tail containing Pro, Glu, Ser, Thr sequences (aa. 352–401) and a postsynaptic-density protein of 95 kDa, discs large, zona occludens-1 domain-interaction motif (aa. 401–403)^6, 7^. PTEN acts as a tumor suppressor by downregulating the cancer-promoting phosphatidylinositol 3-kinase (PI3K)-Akt signaling pathway^6^. Reducing functional PTEN levels is often associated with elevated activities of PI3K and Akt^8^. PTEN is frequently mutated or deleted in tumors including HCC^6, 9^. Two naturally occurring mutations disrupt the phosphatase activities of PTEN: C124S mutation abrogates both lipid and protein phosphatase activity, and G129E mutation abrogates lipid phosphatase activity only. The Y138L mutant has no protein phosphatase activity^10^. Of note, the human hepatoma Huh-7 cells used in HCV research contain a functional PTEN^11, 12^.

It has been reported that HCV activates the PI3K-Akt pathway^13–16^, which in turn enhances HCV entry, translation, and replication^17–20^. However, the effect of PTEN on HCV life cycle has not been well characterized. An early study showed that HCV infection increases PTEN phosphorylation without affecting the total PTEN level^21^. In contrast, another study showed that HCV infection is associated with less nuclear PTEN that favors HCV replication, suggesting a possible role of PTEN in regulating HCV replication^22^. This is supported by additional studies demonstrating that HCV core and NS5A proteins downregulate PTEN protein levels^23–26^. However, a recent report showed that PTEN only downregulates HCV secretion, but not replication^24^. Together, the exact role of PTEN in HCV life cycle is not clear and requires further investigation.

In this study, we showed that PTEN inhibits HCV entry and replication, but has no effect on HCV RNA translation. PTEN interacts with HCV core protein and inhibits HCV replication.

## Results

### PTEN negatively regulates HCV RNA levels after HCV infection

**Figure 1:**
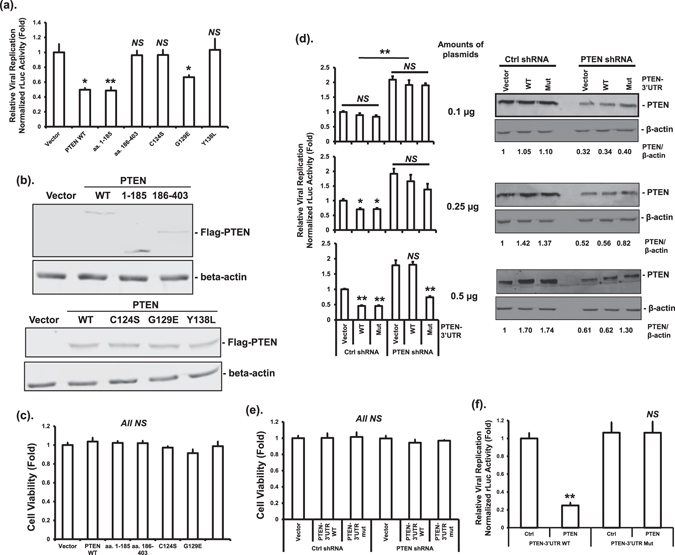
PTEN protein phosphatase activity is required for decreasing HCV RNA level after HCV infection. (**a**) Huh-7.5 cells were transfected with plasmids expressing wild-type Flag-PTEN (PTEN WT), truncated mutants, phosphatase deficient mutants, or empty vector. Forty eight hours after transfection, cells were infected with HCV-2a J6/JFH-1(p7-rLuc-2A) virus at a multiplicity of infection (MOI) of 1. Luciferase assay was performed using the cell lysates 72 hours post-infection (hpi). This experiment was performed three times. (**b**) The protein levels of PTEN proteins and β-actin after transfection were determined by Western blotting using anti-Flag and anti-β-actin antibodies, respectively. (**c**) Cell viability was determined by MTT assay 72 hpi. This experiment was performed three times. (**d**) Huh-7.5 cells expressing an inducible PTEN shRNA or non-silencing control shRNA were treated with 1 μg/mL of Doxycycline for 48 hours. Cells were then infected with HCV-2a J6/JFH-1(p7-rLuc-2A) virus at an MOI of 1. At 24 hpi, cells were transfected with increasing amounts of plasmids expressing Flag-tagged PTEN with wild-type or mutant 3′UTR, or empty vector. Luciferase assay was performed 48 hours after transfection (left panel). In the right panel, the protein levels of PTEN and β-actin at 36 hours after transfection were determined by Western blotting using anti-PTEN and anti-β-actin antibodies, respectively. Relative PTEN band intensities against β-actin were given underneath each sample. This experiment was performed twice. (**e**) Cell viability was determined by MTT assay in parallel with luciferase assay. This experiment was performed twice. (**f**) Huh-7 cells were co-transfected with plasmids expressing non-silencing control shRNA or shRNA targeting the 3′UTR of PTEN, together with bicistronic RNA interference reporter plasmids with (PTEN-3′UTR WT) or without (PTEN-3′UTR Mut) the shRNA target sequence. Dual luciferase assay was performed 48 hours after transfection. The renilla luciferase (rLuc) activity representing the knockdown efficiency was normalized against firefly luciferase activity driven by a constitutive promoter on the reporter plasmid. Luciferase activities were expressed as fold changes relative to vector control. The statistical differences between samples were demonstrated as * if *p* ≤ 0.05, ** if *p* ≤ 0.01, or *NS* for not significant. This experiment was performed three times.

Because the role of PTEN in HCV life cycle is not clear^13–20^, we determined the effect of PTEN on HCV RNA levels after infection with cell culture-derived HCV (HCVcc). Figure 1a showed that overexpression of full-length PTEN (aa. 1–403), or the aa. 1–185 fragment, but not aa. 186–403, significantly reduced HCV RNA levels. Because the aa. 1–185 fragment contains the phosphatase domain of PTEN, these data indicated that the protein and/or lipid phosphatase activities are required for inhibiting HCV RNA accumulation. To test this, we used three phosphatase-deficient mutants. The lipid phosphatase deficient G129E mutant behaved the same as wild-type, whereas the protein phosphatase deficient Y138L, and the dual lipid and protein phosphatase deficient C124S mutants had no effect (Fig. 1a). The expression of wild-type or mutant PTEN proteins, demonstrated by Western blotting (Fig. 1b), had no effects on cell viability as measured by MTT assay (Fig. 1c). These results suggest that PTEN negatively regulates HCV RNA levels after viral infection.

To confirm the inhibitory effects of PTEN on HCV, we determined whether decreasing PTEN enhances HCV RNA levels. We used an inducible shRNA targeting the 3′UTR of PTEN to achieve PTEN knockdown. We first determined the efficiency and specificity of the PTEN shRNA using a bicistronic RNA interference reporter system. PTEN shRNA significantly decreased the luciferase activity linked to wild-type PTEN-3′UTR as compared to a non-silencing control shRNA, indicating an effective knockdown (Fig. 1f). In contrast, the PTEN shRNA did not affect the luciferase activity linked to a mutant PTEN-3′UTR without the targeting sequence of the shRNA. These data indicated that the shRNA can effectively and specifically reduce PTEN levels.

Next, we generated Huh-7.5 cells that express the PTEN shRNA or non-silencing control shRNA upon induction with Doxycycline. Expression of the PTEN shRNA resulted in significantly higher HCV RNA level after HCV infection than control shRNA (Fig. 1d). To demonstrate whether the altered HCV RNA levels detected were specifically due to changes in PTEN levels, the inducible knockdown cell lines were transfected with increasing amounts of plasmids expressing PTEN-3′UTR WT, or shRNA-resistant PTEN-3′UTR Mut. Increasing the levels of PTEN protein after PTEN-3′UTR WT or PTEN-3′UTR Mut transfection in Ctrl shRNA cells was associated with decreasing HCV RNA levels (Fig. 1d). In contrast, increasing the levels of PTEN protein after PTEN-3′UTR WT transfection in PTEN shRNA cells had no effect. Importantly, transfection with PTEN-3′UTR Mut in PTEN shRNA cells decreased HCV RNA levels in a dose-dependent manner (Fig. 1d). The overall PTEN levels in these cells were determined by Western blotting using a PTEN-specific antibody (Fig. 1d). Knocking down PTEN had no effect on cell viability (Fig. 1e). Taken together, these data suggest that PTEN decreases HCV RNA levels after HCV infection in a dose-dependent manner.

Because the HCV RNA levels measured represent the combined effects of PTEN on HCV entry, replication, and translation, we studied the effects of PTEN on each of these steps in the HCV life cycle.

### PTEN lipid phosphatase activity inhibits HCV entry

**Figure 2:**
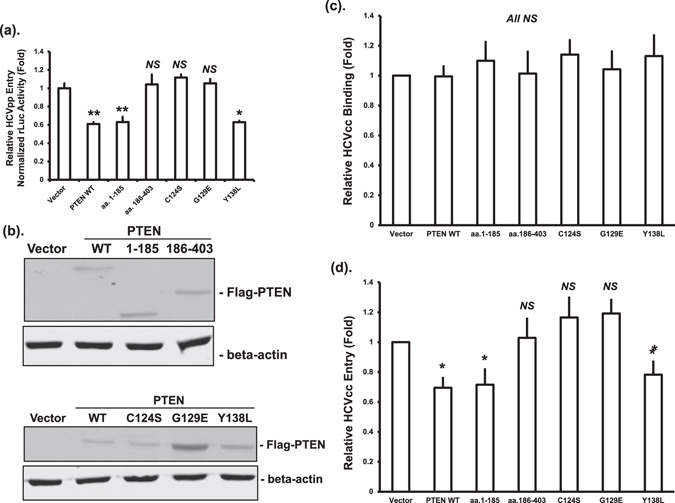
PTEN inhibits HCV entry through its lipid phosphatase activity. (**a**) Huh-7.5 cells were transfected with plasmids expressing wild-type Flag-PTEN (PTEN WT), truncated mutants, phosphatase deficient mutants, or empty vector. Forty eight hours after transfection, cells were infected using HCV-2a J6 pseudoparticles with a firefly luciferase reporter (HCVpp-Luc). Luciferase activity was measured 24 hours after infection and normalized to total protein amount. Luciferase activities were expressed as fold changes relative to vector control. Statistical differences between samples were demonstrated as * if *p* ≤ 0.05, ** if *p* ≤ 0.01, or *NS* for not significant. This experiment was performed three times. (**b**) The protein levels of Flag-tagged PTEN after transfection were determined by Western blotting using an anti-Flag antibody. The β-actin levels were determined by blotting cell lysates with a β-actin antibody as loading controls. (**c** and **d**) Huh-7.5 cells were transfected with plasmids expressing wild-type PTEN (PTEN WT), truncated mutants, phosphatase deficient mutants, or empty vector, and infected with HCV-2a J6 core-Flag/JFH-1 (p7-rLuc-2A) virus 48 hours after transfection. After incubation at 4 °C for 1 hour (**c**, virus binding only) or after a further incubation at 37 °C for 3 hours (**d**, post-binding entry), cells were harvested and RNA extracted. HCV RNA levels were quantified by real-time PCR. Statistical differences between samples were demonstrated as ** if *p* ≤ 0.01, * if *p* ≤ 0.05, or *NS* for not significant.

To study the effect of PTEN on HCV entry, we used the HCVpp approach that we have previously described^27^. Overexpression of full-length PTEN (PTEN WT) and the aa. 1–185 fragment, significantly inhibited HCVpp entry, whereas aa. 186–403 had no effect (Fig. 2a), suggesting a role for the phosphatase activity in this process. To test this, we once again used the three phosphatase-deficient mutants. The protein phosphatase deficient Y138L mutant inhibited HCVpp entry as effectively as the wild-type PTEN, whereas the lipid phosphatase deficient G129E, and the dual lipid and protein phosphatase deficient C124S mutants, had no effect on HCV entry (Fig. 2a). The expression of wild-type or mutant PTEN proteins was confirmed by Western blotting (Fig. 2b). These data collectively indicated that PTEN lipid phosphatase but not protein phosphatase activity is required for HCV entry inhibition.

To extend the above findings, we studied the effects of PTEN on the binding and subsequent entry of HCVcc by determining HCV RNA levels. Overexpression of PTEN or the truncation and phosphatase mutants had no effect on the binding of HCVcc to Huh-7.5 cells in comparison to vector control samples (Fig. 2c). Full-length PTEN and the aa. 1–185 fragment, but not the aa. 186–430 fragment, significantly reduced HCVcc entry (Fig. 2d). When the phosphatase mutants were tested, the protein phosphatase deficient Y138L mutant inhibited HCVcc entry, whereas the lipid phosphatase deficient mutants G129E and C124S had no effect (Fig. 2d). These data indicated that PTEN does not affect the binding of HCV to the cell surface, but can inhibit post-binding entry through its lipid phosphatase activity.

### PTEN protein phosphatase activity is required for the inhibition of HCV replication

**Figure 3:**
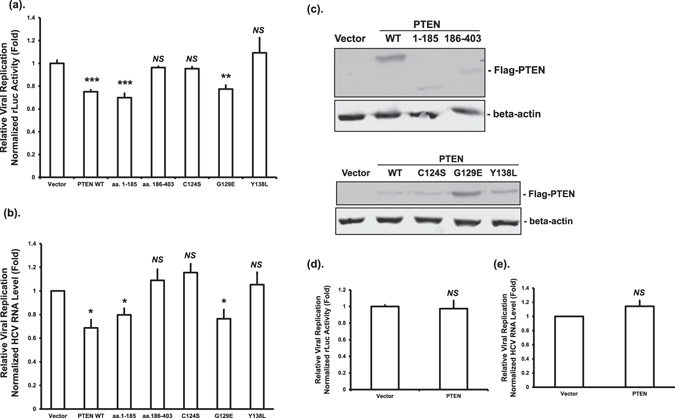
PTEN protein phosphatase activity is required for inhibition of HCV replication in HCV genomic replicon cells. (**a** and **b**) Huh-7 cells with an HCV-2a J6/JFH-1(p7-rLuc-2A) genomic replicon were transfected with plasmids expressing wild-type Flag-PTEN (PTEN WT), truncated mutants, phosphatase deficient mutants, or empty vector. Forty eight hours after transfection, luciferase activities (**a**) or HCV RNA levels (**b**) were determined. (**c**) The protein levels of PTEN and β-actin were determined by Western blotting using anti-PTEN and anti-β-actin antibodies, respectively. (**d** and **e**) Huh-7 cells with an HCV-2a JFH-1 rLuc subgenomic replicon were transfected with plasmid vector or PTEN expressing plasmid. Luciferase (**d**) and HCV RNA levels (**e**) were determined 48 hours after transfection. Data were expressed as fold changes relative to vector control; *p ≤ 0.05, **p ≤ 0.01, ***p ≤ 0.001, or *NS* for not significant.

We next determined the effect of PTEN on HCV replication in HCV genomic replicon cells. The replication of HCV-2a J6/JFH-1(p7-rLuc-2A) RNA was demonstrated by either the luciferase levels or the HCV RNA levels. Overexpression of full-length PTEN, or the aa. 1–185 fragment, but not aa. 186–403, significantly inhibited HCV replication (Fig. 3a and b). These data indicated that the phosphatase domain of PTEN is required for inhibiting HCV replication. Consistently, PTEN with G129E mutation, but not C124S or Y138L mutations, inhibited HCV replication (Fig. 3a and b). PTEN protein expression was confirmed by Western blotting (Fig. 3c). These data suggested that the protein phosphatase activity of PTEN is essential for the inhibition of HCV RNA replication. To study the roles of viral structural and non-structural proteins in HCV replication inhibition by PTEN, we determined the effects of PTEN on the replication of an HCV subgenomic replicon. We found that PTEN did not inhibit HCV replication in the absence of HCV structural proteins (Fig. 3d for luciferase levels and 3e for HCV RNA levels). These data collectively indicated that PTEN inhibits HCV replication with the possible involvement of viral structural proteins.

### PTEN does not affect HCV RNA translation

**Figure 4:**
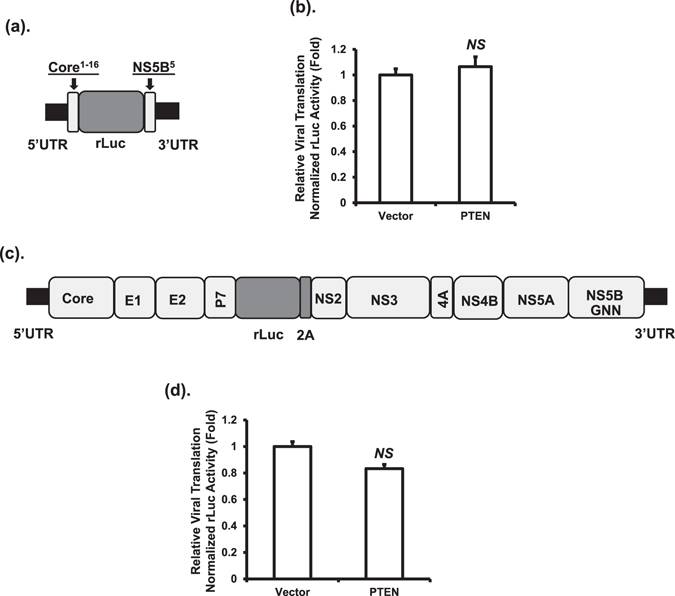
PTEN has no effect on HCV RNA translation. HCV translation was measured using either a monocistronic HCV-2a rLuc reporter (**a**) or a replication-deficient HCV-2a genomic RNA rLuc reporter (**c**). Huh-7 cells were co-transfected with the monocistronic translation rLuc reporter RNA (**b**) or the genomic RNA (**d**), and plasmid vector or PTEN-expressing plasmid. Luciferase assay was performed 24 hours after transfection. Data were expressed as fold changes relative to vector control; *NS* for not significant.

To examine whether PTEN affects HCV RNA translation, we used well-established translation reporters (Fig. 4a and c) that we reported previously^19, 28^. Huh-7 cells were co-transfected with a monocistronic HCV translation rLuc reporter RNA (Fig. 4b) or HCV genomic RNA that is replication-deficient (Fig. 4d), together with a plasmid vector or a PTEN-expressing plasmid. At 24 hours after transfection, the degree of HCV RNA translation was measured by quantification of the luciferase levels. No significant changes in RNA translation were observed in both cases (Fig. 4b and d). These data indicated that PTEN has no effect on HCV RNA translation.

### PTEN interacts with the HCV core protein

**Figure 5:**
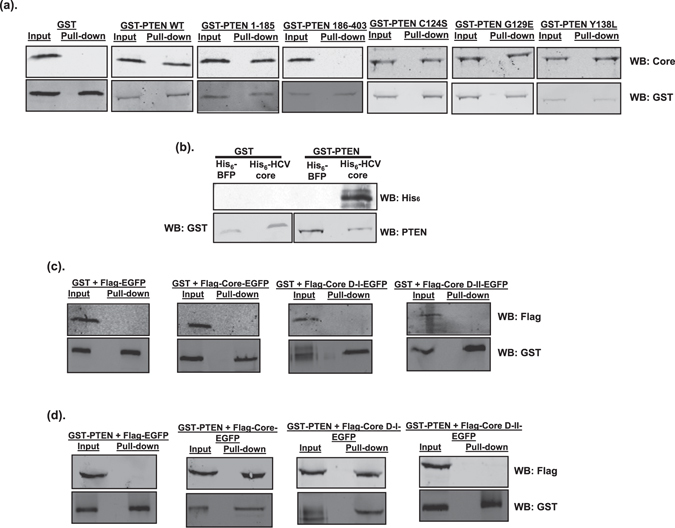
PTEN interacts with the domain I of HCV core. (**a**) Huh-7 cells harboring HCV-2a J6/JFH-1(p7-rLuc-2A) replicon were transfected with plasmids expressing GST, GST-PTEN wild-type or GST-PTEN mutants. At 48 hours after transfection, cell lysates were subjected to GST pull-down assay. Input and pull-down products were analyzed by Western blotting (WB) using anti-HCV core and anti-GST antibodies, respectively. (**b**) Purified His_6_-BFP (blue fluorescent protein), His_6_-HCV core, GST and GST-PTEN proteins were subjected to GST pull-down assay. Pull-down products were analyzed by Western blotting (WB) using anti-His_6_, anti-GST and anti-PTEN antibodies, respectively. Western blot image with a “shorter exposure” can be found in the Supplementary Information. (**c** and **d**) Huh-7 cells were co-transfected with plasmids expressing Flag-tagged HCV core, either full-length, domain I (D-I; aa. 1–118), or domain II (D-II, aa. 119–178), together with plasmids expressing GST (**c**) or GST-PTEN (**d**). The core proteins were expressed as fusion proteins with EGFP. Flag-tagged EGFP-expressing plasmid was included as negative control. At 48 hours after transfection, cell lysates were subjected to GST pull-down assay. Input and pull-down products were analyzed by Western blotting (WB) using anti-Flag and anti-GST antibodies, respectively.

Our results suggested a role for the HCV structural proteins in viral replication inhibition by PTEN (Fig. 3). Among the three HCV structural proteins, the core protein is more likely to play a role in regulating viral replication than the two envelope proteins^4, 29^. In addition, both PTEN and the HCV core proteins often exert their functions through protein - protein interactions^30–32^. Therefore, we investigated whether PTEN interacts with the core protein. HCV genomic replicon cells were transfected with plasmids expressing GST-PTEN or GST, and GST-pull down assay was performed. HCV core protein was present in the pulled down complex by GST-PTEN, but not GST itself, suggesting an association between these two proteins (Fig. 5a). To determine the key regions of PTEN responsible, we used truncated PTEN in the GST pull-down assay. Results showed that PTEN aa. 1–185, but not aa. 186–403, was able to pull down the HCV core protein (Fig. 5a). We also determined whether the phosphatase deficient mutants of PTEN could pull down HCV core. Figure 5a showed that the C124S, G129E, and Y138L mutants were able to pull down HCV core protein, suggesting that these amino acid residues were not involved. These data indicated that the PTEN aa. 1–185 fragment is involved in the association with HCV core protein.

To determine whether PTEN directly interacts with HCV core, His_6_-HCV core and GST-PTEN proteins were purified from *E*. *coli* and used in the GST pull-down assay. Purified His_6_-blue fluorescent protein (BFP) and GST proteins were used as the control proteins. Figure 5b showed that His_6_-HCV core was pulled down by GST-PTEN, but not by GST itself. No His_6_-BFP was detected in the pull down complexes by GST or GST-PTEN. These data indicated that PTEN directly interacts with the HCV core protein.

### HCV core R50 in domain I is the key residue required for the interaction with PTEN

Next, we determined the regions and residues of HCV core required for the interaction with PTEN. Mature HCV core contains two domains, domain I and domain II^33^. To determine which domain of HCV core was required for the interaction with PTEN, Huh-7 cells were co-transfected with plasmids expressing GST-PTEN, and Flag-tagged HCV core fused with enhanced green fluorescent protein (EGFP). Results of the GST pull down assays showed that the full-length core protein, as well as domain I, interacted with PTEN, whereas domain II did not (Fig. 5c). No interactions were detected between proteins involving control proteins EGFP and GST (Fig. 5c and d). These data suggest that domain I of HCV core is required for the interaction with PTEN.

**Figure 6:**
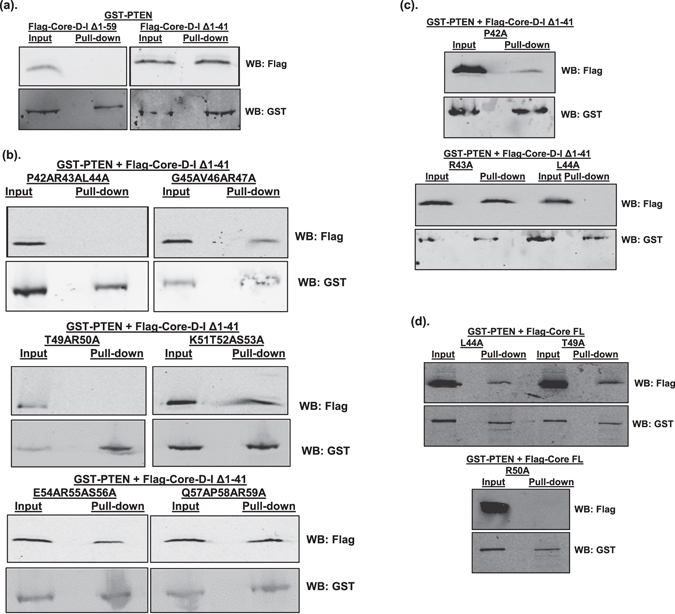
HCV core R50 is the key residue for interaction with PTEN. Huh-7 cells were co-transfected with GST-PTEN-expressing plasmid, together with plasmids expressing truncated HCV core, either wild-type (**a**), with multiple (**b**) or single (**c**) amino acid mutations, or full-length (FL) HCV core with single amino acid mutations (**d**). The core proteins have a Flag-tag at the N-termini. At 48 hours after transfection, cell lysates were subjected to GST pull-down assay. Input and pull-down products were analyzed by Western blotting (WB) using anti-Flag and anti-GST antibodies, respectively. Western blots for Flag-HCV core and GST-PTEN in Fig. 6b were cropped images from full-length blots to ensure lane alignment and the full-length blots can be found in Supplementary Information.

To map the interacting region within domain I (aa. 1–118), we constructed plasmids expressing truncations of domain I and used them in GST pull down assays. We observed that PTEN interacted with HCV core ∆aa. 1–41, but not ∆aa. 1–59 (Fig. 6a). These data indicate that HCV core aa. 42–59 contains the residues necessary for interacting with PTEN. To identify the residue(s) responsible for interacting with PTEN, we employed an alanine scanning approach by mutating groups of two or three amino acid residues at a time into alanines. We found that the P42AR43AL44A and T49AR50A mutants did not interact with PTEN, whereas the G45AV46AR47A, K51AT52AS53A, E54AR55AS56A, and Q57AP58AR59A mutants could associate with GST-PTEN (Fig. 6b). These data suggest that the amino acid residues P42, R43, L44, T49, and/or R50 of HCV core are likely involved in the interaction with PTEN. We therefore substituted each of these residues with alanines individually, and performed GST pull down assays. PTEN interacted with the P42A and R43A mutants, but not the L44A, T49A or R50A mutants (Fig. 6c). These data indicated that, when aa. 1–41 is deleted, the amino acid residues L44, T49 and R50 of HCV core are involved in the interaction with PTEN.

Because the aa. 1–41 of HCV core contains a helix-loop-helix structure as per molecular modeling^34^, deletion of this structure may have an impact on the overall conformation of the HCV core protein and thus affect protein - protein interactions. We therefore tested each of the L44A, T49A and R50A mutations in the context of full-length HCV core and measured the interaction with PTEN. GST pull-down assay results showed that the full-length L44A and T49A mutant core proteins could still interact with PTEN, whereas the R50A mutation abolished the interaction completely (Fig. 6d). Taken together, these data indicated that R50 of HCV core domain I is the key residue required for the interaction with PTEN.

### HCV core interacts with endogenous PTEN through R50

**Figure 7:**
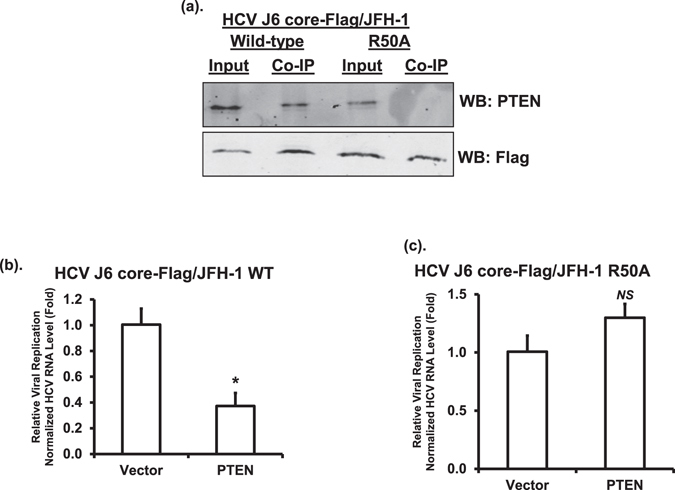
PTEN-HCV core interaction in HCV replicating cells and inhibits HCV replication. (**a**) The lysates prepared from Huh-7 cells harboring HCV-2a J6 core-Flag/JFH-1(p7-rLuc-2A) or HCV-2a J6 core R50A-Flag/JFH-1(p7-rLuc-2A) replicating RNAs were subjected to immunoprecipitation (IP) using an anti-Flag antibody. Input and IP products were analyzed by Western blotting (WB) using anti-PTEN and anti-HCV core antibodies, respectively. This experiment was performed twice. (**b** and **c**) Huh-7 cells harboring HCV-2a J6 core-Flag/JFH-1(p7-rLuc-2A) (**b**) or HCV-2a J6 core R50A-Flag/JFH-1(p7-rLuc-2A) (**c**) replicating RNAs were transfected with vector or PTEN-expressing plasmid. At 48 hours after transfection, HCV RNA level was determined by reverse transcription real-time PCR using HCV-2a-specific primers. The transcript level of β-glucuronidase (GUSB) was used for normalization. The statistical differences between samples were demonstrated as * if *p* ≤ 0.05, ** if *p* ≤ 0.01, or *NS* for not significant. This experiment was performed twice.

Data generated so far had shown an interaction between HCV core and PTEN after ectopic expression. To demonstrate whether HCV core interacts with endogenous PTEN in HCV replicating cells, we generated Huh-7 cells harboring HCV-2a J6 core-Flag/JFH-1(p7-rLuc-2A) replicating RNA. The addition of a Flag tag to the core sequence can be tolerated by HCV^35^ and allows convenient co-immunoprecipitation (co-IP) using a Flag-specific antibody. Figure 7a showed that PTEN was present in the immunoprecipitates with anti-Flag antibody, suggesting that HCV core interacts with the endogenous PTEN protein in HCV replicating cells. To determine the role of R50 of HCV core in this interaction, we also generated Huh-7 cells harboring HCV-2a J6 core R50A-Flag/JFH-1(p7-rLuc-2A) replicating RNA. PTEN protein was not detected in the anti-Flag immunoprecipitates using the R50A lysates (Fig. 7a). These data demonstrated that HCV core R50 is the key residue for the interaction with endogenous PTEN in HCV replicating cells.

### PTEN - HCV core interaction is required for inhibiting HCV replication by PTEN

To determine the role of PTEN - core interaction in regulating HCV replication, HCV RNA levels were quantified in wild-type and R50A HCV replicating cells after overexpression of PTEN. We observed that overexpression of PTEN inhibited HCV replication in wild-type, but not in R50A, HCV replicating cells (Fig. 7b a﻿nd c). These data indicated that the inhibition of HCV replication by PTEN requires PTEN - core interaction.

### PTEN - core interaction is associated with reduced PTEN levels and intracellular lipid abundance

**Figure 8:**
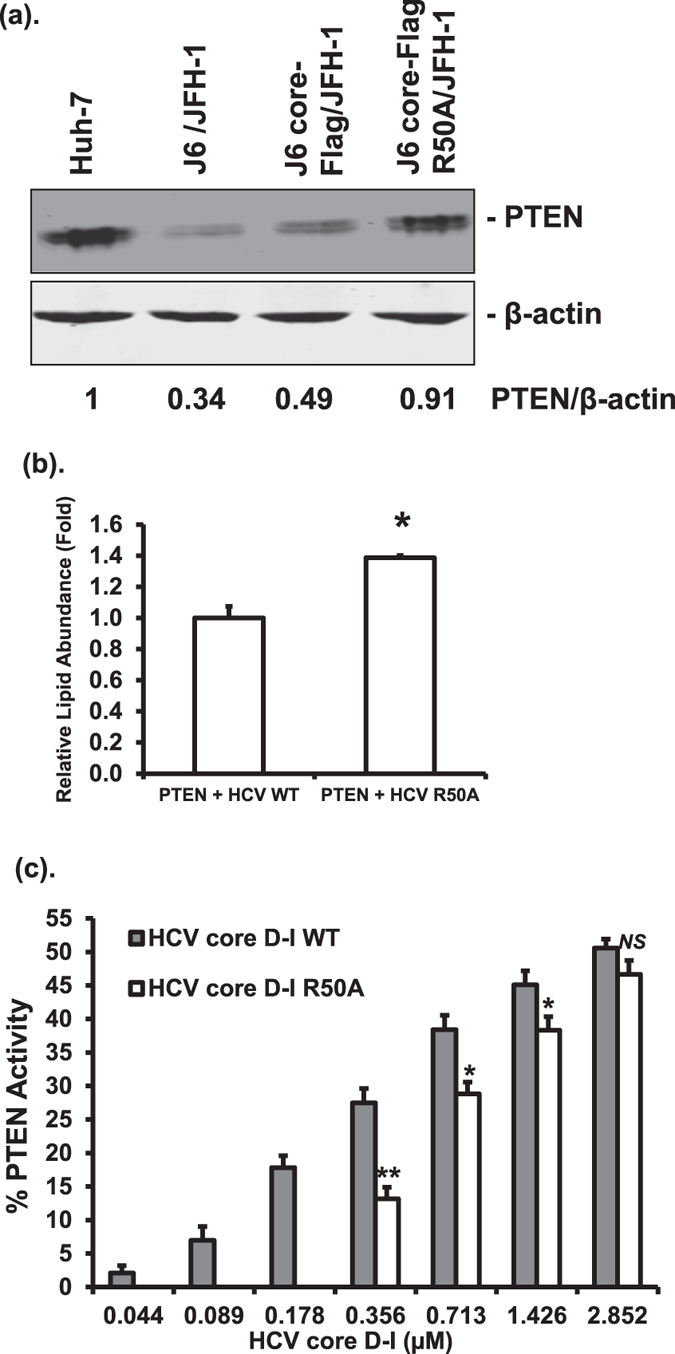
PTEN-HCV core interaction reduces PTEN levels, intracellular lipid abundance, and increases lipid phosphatase activity of PTEN. (**a**) The protein levels of PTEN in parental Huh-7 cells, Huh-7 cells harboring HCV-2a J6/JFH-1(p7-rLuc-2A), HCV-2a J6 core-Flag/JFH-1(p7-rLuc-2A) or HCV-2a J6 core-Flag R50A/JFH-1(p7-rLuc-2A) were determined by Western blotting using anti-PTEN antibody. The levels of β-actin were also determined using anti-β-actin antibody. Relative PTEN band intensities against β-actin were given underneath each sample. This experiment was performed three times. (**b**) Huh-7 cells harboring HCV-2a J6/JFH-1(p7-rLuc-2A) (HCV WT) or HCV-2a J6 core-Flag R50A/JFH-1(p7-rLuc-2A) (HCV R50A) RNAs were transfected with a plasmid expressing PTEN. At 48 hours after transfection, cells were subjected to Oil Red O staining and neutral lipids were quantified. The statistical difference between samples was demonstrated as * if *p* ≤ 0.05. (**c**) Purified PTEN protein (20 nM) was incubated with increasing amounts of HCV core domain I (HCV core D-I) proteins, wild-type or R50A, as indicated. The phosphatase activity of PTEN towards PtdIns(3,4,5)P_3_ was determined in the presence of increasing concentrations of the HCV core D-I proteins and presented relative to PTEN alone (set to a value of 1). The statistical differences between samples were demonstrated as * if *p* ≤ 0.05, ** if *p* ≤ 0.01, or *NS* for not significant. This experiment was performed three times.

Since previous studies demonstrated that HCV core can reduce PTEN levels^23, 26^, we were interested in investigating whether the interaction between PTEN and HCV core can also regulate PTEN levels as a mechanism of modulating HCV RNA replication. We therefore determined the amounts of PTEN in the HCV RNA replicating cells by Western blotting. As shown in Fig. 8a, PTEN protein levels in Huh-7 cells harboring wild-type replicating HCV RNA were lower than those in Huh-7 cells harboring HCV R50A replicating RNA and in the parental Huh-7 cells. These results suggested that the PTEN protein level is reduced in HCV replicating cells most likely due to its interaction with HCV core.

Because HCV infection and HCV core protein are known to up-regulate host lipogenesis that favors HCV replication^36, 37^, we wanted to study whether PTEN - core interaction modulates intracellular lipid abundance. Quantification of intracellular neutral lipids in HCV replicating cells after transfection with a PTEN-expressing plasmid indicated that the lipid abundance in mutant HCV R50A replicating cells was significantly higher than that in the wild-type HCV replicating cells (Fig. 8b). These data suggested that more lipids are accumulated in the absence of PTEN - core interaction, which in turn may abrogate the inhibitory effect of PTEN on HCV replication.

### HCV core domain I increases the lipid phosphatase activity of PTEN

Data presented so far had demonstrated that the phosphatase activities of PTEN are required for its inhibitory effects on HCV. In addition, we showed that the interaction between HCV core and PTEN is involved in inhibiting HCV replication by PTEN. Because the phosphatase activities of PTEN can be modulated upon interacting with a protein^38^, we wanted to determine whether HCV core - PTEN interaction has an effect on the phosphatase activity of PTEN. We employed an enzymatic malachite green assay to measure the lipid phosphatase activity of PTEN after incubating with HCV core protein. Because we encountered some difficulty in obtaining the full-length core protein in the quantity and purity required for the assay, we instead used HCV core domain I protein, the domain responsible for PTEN interaction. To determine the effect of HCV core - PTEN interaction on the phosphatase activity of PTEN, we also used HCV core domain I R50A mutant protein in the phosphatase assay. Lipid phosphatase activity of PTEN at 20 nM was measured after incubating with increasing amounts of HCV core domain I proteins, either wild-type or with the R50A mutation. As shown in Fig. 8c, in the presence of the wild-type HCV core domain I protein at the three lowest concentrations tested, there was a steady increase in the lipid phosphatase activity of PTEN, whereas no activity was measurable in the presence of the R50A protein. With increasing amounts of the core domain I proteins, the lipid phosphatase activity of PTEN also increased in both cases. Except for the highest concentration tested, incubation with the wild-type core protein resulted in significantly higher lipid phosphatase activity of PTEN than the R50A protein. These data indicated that the presence of HCV core domain I protein increases the lipid phosphatase activity of PTEN with the wild-type core being more effective than the R50A mutant core.

## Discussion

Although the effects of the PI3K-Akt signaling pathway on the HCV life cycle are well documented^17–20^, the role of PTEN is less well defined. A previous study showed that PTEN can inhibit HCV virion secretion^24^. As an extension to this study, we showed that PTEN inhibited HCV entry and replication through its different PTEN phosphatase activities. Furthermore, HCV core interacts with PTEN and this interaction regulates the steady level and the lipid phosphatase activity of PTEN as well as intracellular lipid abundance.

We first determined the HCV RNA levels after HCV infection upon ectopic expression of PTEN protein. We showed an approximately 50% reduction in HCV RNA levels after PTEN overexpression with no measurable effects on cell viability (Fig. 1). Moreover, the regulation of HCV RNA levels was confirmed by decreasing PTEN levels by PTEN-specific shRNA. More importantly, the inhibitory effects of PTEN on HCV RNA levels were dose-dependent and PTEN-specific (Fig. 1). We noticed a smaller degree of HCV replication inhibition in PTEN shRNA cells than in control shRNA cells after transfection with the same amounts of PTEN-expressing plasmids (Fig. 1d). In contrast to a significant inhibition of HCV RNA levels in control shRNA cells, the decrease in HCV RNA levels after transfection with 0.25 µg of PTEN-3′UTR Mut in PTEN shRNA cells was not statistically different from that after transfection with 0.1 µg of the plasmid. Further increase of the PTEN-3′UTR Mut plasmid to 0.5 µg resulted in significantly lower HCV RNA levels. This could be explained by lower overall PTEN levels in PTEN shRNA cells than those in control shRNA cells (Fig. 1d).

We next studied how PTEN affected HCV RNA levels. Virus binding and entry is the first step in the HCV life cycle. PTEN reduced HCV entry at the post-binding step through its lipid phosphatase activity, while no effect on HCV binding was observed (Fig. 2). As for replication, PTEN inhibited the replication of HCV genomic RNA, but not subgenomic RNA (Fig. 3). These results suggested a role for viral structural proteins in replication inhibition by PTEN. We noticed a smaller degree of inhibition by PTEN on HCV replication in genomic replicon cells (Fig. 3) in comparison to that after HCV infection (Fig. 1). While the reasons leading to this difference are not clear, we thought this might be primarily due to the fact that HCV RNA levels, as measured in HCV replicon cells, should only represent the degree of HCV RNA replication inhibition by PTEN, whereas those measured after HCV infection should reflect the inhibitory effects of PTEN on both HCV entry and replication. A previous study found that overexpression of PTEN did not have a significant effect on HCV replication using an HCV Jc1 virus^24^. The reasons for the discrepancy are not clear and whether it is due to different experimental conditions or viral strains should be investigated.

We recently demonstrated that the PI3K-Akt signaling pathway upregulates HCV RNA translation^19^. Interestingly however, no effect of PTEN on HCV translation was observed in the current study (Fig. 4). This is not entirely unexpected though because there are examples where the phenotypes of PTEN (down-regulation) may not always result in altered Akt (up-regulation)^6, 39^. Our results suggest that PI3K-Akt modulates HCV RNA translation through different mechanisms than PTEN, which warrants further investigation.

To understand how PTEN inhibits HCV genomic RNA replication, but not subgenomic RNA replication, we sought to determine whether PTEN interacted with the HCV core protein. The reason for us to focus on the core protein, but not the two envelope E1 and E2 proteins, was because the core protein has been shown to exert its regulatory functions through protein - protein interactions and also be involved in regulating PTEN^4, 23, 26, 29^. We indeed found that PTEN interacted with HCV core protein (Fig. 5). Furthermore, we identified the R50 residue within domain I of HCV core protein as the key residue required for mediating this interaction (Fig. 6). More importantly, when R50 was mutated, PTEN could no longer inhibit HCV replication (Fig. 7c), suggesting that this interaction has an important function. Previous studies have shown that the R50 residue of the HCV core protein is involved in interacting with the NS5A protein and is required for HCV infectivity^35, 40^. Our results have demonstrated an additional function for residue R50 in regulating HCV replication by interacting with PTEN, a host cellular protein.

Previous studies have demonstrated that HCV core decreases PTEN level through either blocking PTEN mRNA translation^23^ or activation of NF-κB^26^. We found that the replication of wild-type HCV was associated with decreased PTEN protein level, which was restored to a significant extent when HCV core residue R50 was mutated (Fig. 8a). These data suggest that HCV core - PTEN interaction results in a decrease in the steady level of PTEN. The exact mechanisms of how HCV core - PTEN interaction regulates PTEN levels are not known. It has been shown that PTEN can be modified by ubiquitination and degraded upon interaction with an E3 ubiquitin ligase WWP2^41^. HCV core protein can also interact with and regulate the ubiquitination system^42, 43^. It is possible that the interaction between PTEN and HCV core decreases PTEN levels by enhancing its ubiquitination. This possibility should be investigated in future studies.

Host lipid biogenesis plays a very important role in regulating HCV replication and HCV infection and HCV proteins including the core protein can up-regulate intracellular lipid contents during its life cycle^36, 37^. We found that PTEN - core interaction in HCV replicating cells resulted in reduced lipid levels in comparison to that when there was no such interaction (Fig. 8b). This observation may point to another mechanism by which PTEN modulates HCV replication.

Protein - protein interactions are important in modulating the phosphatase activity of PTEN^38^. We found that the presence of the HCV core domain I protein increased the lipid phosphatase activity of PTEN in a dose-dependent manner in an *in vitro* lipid phosphatase assay (Fig. 8c). In contrast, the HCV core domain I R50A mutant protein was only able to enhance the lipid phosphatase activity at high concentrations tested (Fig. 8c). We noticed a sudden rise in PTEN phosphatase activity when the R50A protein was increased from 0.178 µM to 0.356 µM and further dose-dependent increase at even higher concentrations (Fig. 8c). The mechanism by which this occurred was not clear. Whether this was due to non-specific protein - protein interaction at high concentrations should be studied further. Nevertheless, these results indicated that HCV core - PTEN interaction plays a role in the regulation of PTEN activities, suggesting that HCV infection may also modulate the activity of PTEN.

A primary function of the lipid phosphatase activity of PTEN is downregulating the PI3K-Akt signaling pathway^6^. Since the PI3K-Akt pathway has been shown to promote HCV entry^20^, it is possible that the elevated lipid phosphatase activity of PTEN upon interacting with HCV core plays a role in inhibiting HCV entry, consistent with our entry data (Fig. 2). However, it should be pointed out that the lipid phosphatase activity of PTEN was measured in an *in vitro* assay using purified proteins (Fig. 8c). Given the fact that the PTEN levels were reduced in the presence of HCV core *in vivo* (Fig. 8a), whether the HCV core - PTEN interaction resulted in a net increase in lipid phosphatase activity of PTEN *in vivo* should be studied further.

In summary, we presented evidence to support an inhibitory role of PTEN in regulating HCV entry and replication. This suggests that non-HCV infected liver cancer patients with reduced PTEN levels could be more susceptible to HCV infection^44^. Mechanistically, we demonstrated that HCV core interacts with PTEN and may modulate the functions of PTEN through multiple mechanisms. Our results should help develop additional therapeutics for HCV infection and the resultant hepatocellular carcinoma.

## Methods

### Plasmid constructs and *in vitro* transcription

Plasmids for generating lentiviral particles, pMD2.G, psPAX2, pCMV-HCV-2a J6 core-E1-E2, and pTRIP-CMV-Luc-puro, were described previously^15, 27^. The pGIPZ lentiviral plasmid expressing PTEN-specific shRNA with the target sequence 5′-GAGACAGACTGATGTGTAT-3′ located in the 3′UTR of PTEN mRNA and non-silencing control shRNA were purchased from Open Biosystems. Inducible PTEN shRNA was constructed by transferring the shRNA sequence into a Tet-On pTRIPZ lentiviral vector (Open Biosystems). Plasmid expressing PTEN with an N-terminal Flag-tag, pCMV5 Flag-Human PTEN, was received from Dr. Jack Dixon^45^ and used to generate plasmids expressing truncations and point mutations of PTEN: aa. 1–185, aa. 186–403, C124S, G129E, and Y138L^24^. We also generated PTEN-expressing plasmids with a 3xFlag-tag or GST fusion in the pEF-cyto-myc vector for use in some experiments. To allow the expression of the PTEN protein from the wild-type and shRNA-sensitive transcript, the 3′UTR sequence of the PTEN mRNA, 1000 bps in length, was amplified by reverse transcription - PCR using RNA extracted from Huh-7 cells and cloned 3′ to the coding sequence of PTEN, generating plasmid PTEN-3′UTR WT. The target sequence of the shRNA was then removed from the 3′UTR by site-directed mutagenesis, resulting in an shRNA-resistant PTEN-expressing plasmid PTEN-3′UTR Mut. The wild-type and mutant 3′UTR sequences were also cloned into the RNA interference vector psiCHECK-2, a gift from Dr. Robert Blelloch (Addgene plasmid # 31882)^46^. Plasmids expressing GST-PTEN fusion proteins in the pGEX-6P-1 vector were described previously^47^. Plasmid pFLneo-J6/JFH-1(p7-rLuc-2A) containing the full-length HCV-2a J6/JFH-1 genomic sequence with an internal renilla luciferase reporter gene was obtained from Dr. Charles Rice^48^. Plasmid HCV-2a J6 core-Flag/JFH-1(p7-rLuc-2A) was constructed by insertion of a Flag-tag between the amino acids S2 and T3 of the core protein as previously described^35^. Plasmid HCV-2a J6 core-R50A Flag/JFH-1(p7-rLuc-2A) contains the R50A mutation. A subgenomic replicon plasmid pSGR-rLuc-Neo-HCV-2a JFH-1 NS3–5B with rLuc reporter was constructed as previously described^49^. A monocistronic HCV RNA translation reporter pT7 HCV-2a JFH-1 5′UTR-core^aa1-16^-Luc2-NS5B^5^-3′UTR was constructed as described^28^ using the JFH-1 sequence from the plasmid pHCV-2a JFH-1_pUC provided by Dr. Takaji Wakita^50^. This reporter contains HCV 5′UTR, sequence encoding the N-terminal 16 amino acids of the core protein, an internal rLuc gene, sequence encoding the last five amino acids of the NS5B protein and HCV 3′UTR (Fig. 2a). We also used replication-deficient HCV genomic RNA *in vitro* transcribed from the plasmid pFLneo-J6/JFH-1(p7-rLuc-2A) GNN^48^ to measure HCV RNA translation (Fig. 2c). To generate a plasmid expressing HCV core protein, the core coding sequence was amplified by PCR using the plasmid pFLneo-J6/JFH-1(p7-rLuc-2A) as template and cloned into the pEF vector. Plasmids expressing the individual domains (as EGFP fusions), truncations, or point mutations of HCV core were generated. To allow protein expression in *E*. *coli*, the coding sequences for HCV core, full-length or domain I with or without R50A mutation, were cloned into the pT7 His_6_-SUMO vector (Lucigen). BFP-expressing plasmid in the pT7 His_6_-SUMO vector was described previously^51^. All plasmids were constructed using standard methods and confirmed by DNA sequencing. HCV genomic RNAs and translation reporter RNAs were generated by *in vitro* transcription using the MEGAscript T7 *In Vitro* Transcription reagents (Ambion).

### Cell culture, transfection, and generation of stable cell lines

Huh-7 cells, Huh-7.5 cells and HEK293T cells were cultured in Dulbecco’s modified Eagle’s medium (DMEM) with 10% (v/v) fetal bovine serum (FBS). Cells were transfected with plasmid DNA or HCV RNA using the calcium phosphate precipitation method^52^ or the DMRIE-C reagent^28^ (Thermo Fisher Scientific), respectively. For establishing HCV replicating cells, Huh-7 cells were transfected with HCV RNA and selected by G418 (Enzo Life Sciences)^15^.

### Luciferase, MTT and Oil Red O staining assays

For the luciferase assay, cells were lysed in Passive Lysis Buffer (Promega) and the firefly or renilla luciferase activities were measured by Luciferase Assay reagents (Promega) in a TD 20/20 Luminometer (Turner Designs). Luciferase levels were normalized to the protein concentrations determined by a Bradford assay (Bio-Rad Laboratories). Cell viability MTT assay was performed as previously described^28^. The amounts of the neutral lipids were measured at 500 nm^53^ after Oil Red O (ORO) staining as we previously described^52, 54^.

### RNA extraction and real-time reverse transcription-polymerase chain reaction

RNA was isolated from cells with TriZol (Thermo Fisher Scientific) followed by DNase I (Thermo Fisher Scientific) digestion. Reverse transcription was carried out by Superscript II (Thermo Fisher Scientific) and random priming. HCV RNA levels were determined by real-time PCR using HCV-specific primers HCV-FD (5′-AGAGCCATAGTGGTCTGCGGAAC-3′) and HCV-rev (5′-CCTTTCGCAACCCAACGCTACTC-3′)^55^. The transcript levels of GUSB, a house keeping gene, were used for normalization^56^. Relative changes in RNA levels were analyzed by the 2^−∆∆ct^ method using the iQ5 program (Bio-Rad Laboratories).

### HCV entry, replication, and translation assays

HCV entry was determined using HCV lentiviral pseudoparticles carrying the luciferase reporter gene (HCVpp-Luc) as we previously described^27^. In another set of experiments, Huh-7.5 cells were infected with cell culture-derived HCV-2a J6 core-Flag/JFH-1 (p7-rLuc-2A) virus. Cells were harvested either after incubation at 4 °C for 1 hour (virus binding) or after a further incubation at 37 °C for 3 hours (post-binding entry) as per an established protocol^57, 58^. RNA was extracted and used in a real-time PCR assay. Replication of HCV-2a J6/JFH-1 (p7-rLuc-2A) RNA in Huh-7 cells harboring HCV replicating RNA or after infection with the HCV-2a J6/JFH-1 (p7-rLuc-2A) virus was analyzed by determining the rLuc activity using a luciferase assay or HCV RNA levels using real-time PCR. HCV-2a J6/JFH-1 (p7-rLuc-2A) virus was collected from the supernatant of Huh-7 cells harboring HCV-2a J6/JFH-1 genomic RNA and titrated by focus forming assay as described^48^. HCV RNA translation assay was performed as previously described^19^.

### GST pull-down, co-immunoprecipitation (co-IP), and Western blotting

The GST pull-down assay was performed using cell lysates in radioimmunoprecipitation assay (RIPA) buffer and Glutathione Sepharose 4B (GE Healthcare) according to a standard protocol^47^. For Flag co-IP experiments, cells were harvested in RIPA buffer and incubated with an anti-Flag (Sigma-Aldrich) antibody at 4 °C overnight and then incubated with Protein G Sepharose (GE Healthcare) at 4 °C for 4 hours. The mixtures were centrifuged at 10,000 rpm for 10 minutes and the supernatants removed. The pellets were resuspended in a lysis buffer containing SDS and boiled for 10 minutes to elute the proteins. For Western blotting, proteins were subjected to SDS-PAGE and then blotted onto nitrocellulose membranes. The membranes were blocked in 5% skim milk in PBS and incubated with a primary antibody overnight at 4 °C. Membranes were washed and incubated with a secondary antibody for 1 hour at room temperature. After a wash with PBST (PBS + 0.1% Tween 20), membranes were scanned using a Li-Cor Odyssey scanner (ODY-CLx) and band intensities were determined using the Quantity One software (Bio-Rad Laboratories). The antibodies used were: HCV core (Anogen), GST (Cell Signaling Technology, CST), PTEN (CST), β-actin (CST), Flag (Sigma-Aldrich), His_6_-tag (Roche), and secondary antibodies IRDye 800 CW goat anti-mouse IgG and IRDye 680 RD goat anti-rabbit IgG (Li-Cor Biosciences).

### Purification of recombinant proteins

Expression of recombinant proteins in *E*. *coli* was induced using IPTG (Thermo Fisher Scientific). GST-PTEN fusion proteins were purified using Glutathione Sepharose 4B (GE Healthcare). For use in PTEN phosphatase assay, the PTEN protein was cleaved from GST using the PreScission protease (GE Healthcare) as previously described^59^. His_6_-tagged HCV core and PTEN proteins were purified using Ni-NTA agarose (Qiagen).

### PTEN lipid phosphatase activity assay

The lipid phosphatase activity of PTEN in the presence of HCV core domain I protein was measured using the PTEN Activity ELISA kit (Echelon Biosciences) according to manufacturer’s instructions. Briefly, 20 nM of PTEN was incubated with 0–2.852 µM of HCV core domain I proteins, wild-type or R50A mutant, at 37 °C for 5 minutes prior to the addition of 16 µM of the PtdIns(3,4,5)P_3_ substrate at 37 °C for 1 hour. Activity was detected using a SpectraMax M5 spectrophotometer (Molecular Devices) at 450 nm and presented as the percentage increases in the presence of HCV core domain I proteins compared to PTEN alone.

### Statistical analysis

Experimental results were analyzed for statistical differences by Student’s *t* test. A *p* value of ≤0.05 was considered statistically significant.

## Electronic supplementary material

Supplementary info
