# Supplementary material for: The role of PTEN - HCV core interaction in hepatitis C virus replication
Source: Sci Rep. 2017 Jun 16;7:3695. doi: 10.1038/s41598-017-03052-w (PMC5473856; doi:10.1038/s41598-017-03052-w)

**Supplementary Information**

The role of PTEN - HCV core interaction in hepatitis C virus replication

Qi Wu, Zhubing Li, Paul Mellor, Yan Zhou, Deborah H. Anderson, Qiang Liu

**Figure 5b. “Shorter exprosure”**

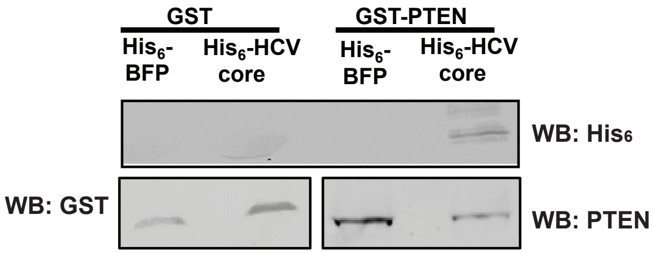


**Figure 5b. “Longer exprosure”**

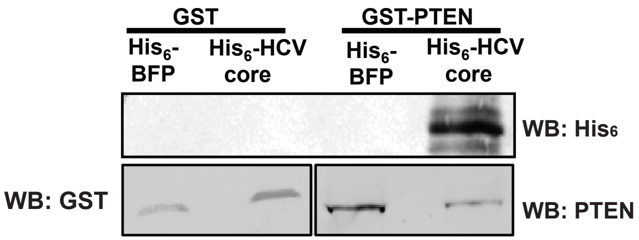


**Figure 6b. Blots prior to cropping**

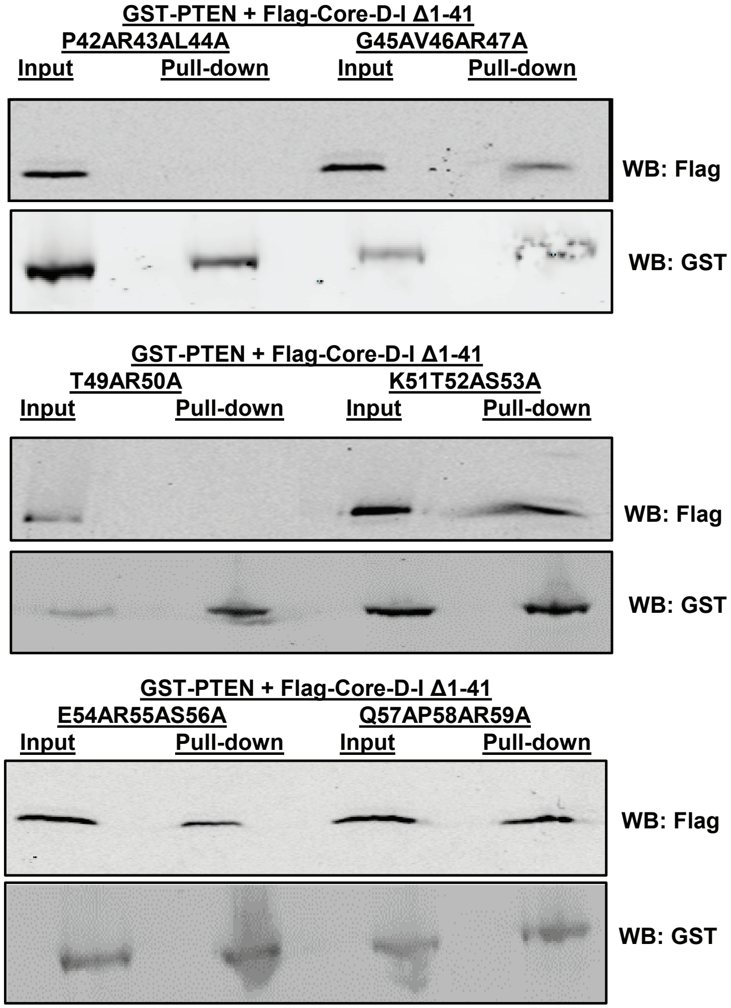

Supplement: Supplementary file 1 — Supplementary info [file 41598_2017_3052_MOESM1_ESM.pdf]
